# Therapeutic potential of nanoceria pretreatment in preventing the development of urological chronic pelvic pain syndrome: Immunomodulation via reactive oxygen species scavenging and SerpinB2 downregulation

**DOI:** 10.1002/btm2.10346

**Published:** 2022-06-13

**Authors:** Wei‐Chih Lien, Xin‐Ran Zhou, Ya‐Jyun Liang, Congo Tak‐Shing Ching, Chia‐Yih Wang, Fu‐I Lu, Huei‐Cih Chang, Feng‐Huei Lin, Hui‐Min David Wang

**Affiliations:** ^1^ Department of Physical Medicine and Rehabilitation National Cheng Kung University Hospital, College of Medicine, National Cheng Kung University Tainan Taiwan, Republic of China; ^2^ Department of Physical Medicine and Rehabilitation, College of Medicine National Cheng Kung University Tainan Taiwan, Republic of China; ^3^ Ph.D. Program in Tissue Engineering and Regenerative Medicine National Chung Hsing University Taichung City Taiwan, Republic of China; ^4^ Institute of Biomedical Engineering, College of Medicine and College of Engineering National Taiwan University Taipei Taiwan, Republic of China; ^5^ Graduate Institute of Biomedical Engineering National Chung Hsing University Taichung City Taiwan, Republic of China; ^6^ Department of Cell Biology and Anatomy, College of Medicine National Cheng Kung University Tainan Taiwan, Republic of China; ^7^ Institute of Basic Medical Sciences, College of Medicine National Cheng Kung University Tainan Taiwan, Republic of China; ^8^ Department of Biotechnology and Bioindustry Sciences, College of Bioscience and Biotechnology National Cheng Kung University Tainan Taiwan, Republic of China; ^9^ The iEGG and Animal Biotechnology Center National Chung Hsing University Taichung City Taiwan, Republic of China; ^10^ Institute of Biomedical Engineering and Nanomedicine National Health Research Institutes Zhunan, Miaoli Taiwan, Republic of China; ^11^ Graduate Institute of Medicine, College of Medicine Kaohsiung Medical University Kaohsiung Taiwan, Republic of China; ^12^ Department of Medical Laboratory Science and Biotechnology China Medical University Taichung City Taiwan, Republic of China

**Keywords:** cerium oxide nanoparticle, immunomodulation, SerpinB2, urological chronic pelvic pain syndrome

## Abstract

Urological chronic pelvic pain syndrome (UCPPS) manifests as pelvic pain with frequent urination and has a 10% prevalence rate without effective therapy. Nanoceria (cerium oxide nanoparticles [CNPs]) were synthesized in this study to achieve potential long‐term pain relief, using a commonly used UCPPS mouse model with cyclophosphamide‐induced cystitis. Transcriptome sequencing analysis revealed that serpin family B member 2 (SerpinB2) was the most upregulated marker in mouse bladder, and SerpinB2 was downregulated with CNP pretreatment. The transcriptome sequencing analysis results agreed with quantitative polymerase chain reaction and western blot analysis results for the expression of related mRNAs and proteins. Analysis of Gene Expression Omnibus (GEO) datasets revealed that SerpinB2 was a differentially upregulated gene in human UCPPS. In vitro SerpinB2 knockdown downregulated proinflammatory chemokine expression (chemokine receptor CXCR3 and C‐X‐C motif chemokine ligand 10) upon treatment with 4‐hydroperoxycyclophosphamide. In conclusion, CNP pretreatment may prevent the development of UCPPS, and reactive oxygen species (ROS) scavenging and SerpinB2 downregulation may modulate the immune response in UCPPS.

## 1 INTRODUCTION

Urological chronic pelvic pain syndrome (UCPPS), a debilitating condition of chronic visceral pain, is characterized by chronic lower abdominal and pelvic pain, and frequent and urgent urination, which causes disability and has an unknown etiology.^1^ Autoimmune processes, an inadequate glycosaminoglycan layer constitution, unspecified infection, toxic urinary constituents, and central neurogenic processes are some of the pathophysiological factors found in UCPPS.^1^ Chronic inflammation and serial induction play integral roles in UCPPS progression.^2^, ^3^ UCPPS is prevalent in approximately 10% of the general US population.^4^ UCPPS constitutes a significant socioeconomic impact of more than US$ 70 billion annually, while the treatment costs for UCPPS accelerate at a compound annual growth rate of approximately 5%. In addition to healthcare costs, UCPPS negatively influences psychosocial health, causing mental health issues such as sleep disturbance, anxiety, and depression.^5^

The cyclophosphamide (CYP)‐induced chronic cystitis mouse model, where mice are exposed to fourfold serial low‐dose CYP induction, is the most commonly used mouse model to study UCPPS according to the Multidisciplinary Approach to the Study of Chronic Pelvic Pain (MAPP) Research Network.^3^ The etiologies of visceral pain, in contrast to awareness of somatic pain, remain inadequately understood, and the various states of chronic visceral pain are troublesome for efficient treatment. Cytokines and cytokine‐related proteins, including the C‐C motif ligand family and C‐X‐C motif ligand (CXCL) family, play important roles in the pathogenesis of chronic visceral pain, including UCPPS. The distinctive effects of CXCL9 and CXCL10 on pain may be related to the fact that CXCL10 only upregulates the biological process of excitatory synaptic transmission, whereas CXCL9 upregulates the natural functions of both excitatory and inhibitory synaptic transmission.^6^ Clinical studies evaluating patients with various inflammatory or painful conditions involving pelvic organs and animal models of visceral inflammation or pain designate a role for chemokines at the beginning or during the perpetuation of visceral inflammation or pain. A stoppage of CXCL10 signaling diminishes the severity of experimental autoimmune cystitis by reducing the infiltration of inflammatory cells and the expression of pro‐inflammatory chemokines and cytokines in the urinary bladders of mice.^7^

The efficacy of noninvasive or minimally invasive therapies (such as local injections, intravenous injections in humans, or intraperitoneal injections in mice), including physical and pharmacological therapy, is limited by its duration. Thus, novel noninvasive and minimally invasive treatments are needed to manage UCPPS. Pentosan polysulfate sodium, sold under the brand name Elmiron®, is a Food and Drug Administration‐approved orally administered drug used for the treatment of UCPPS. Elmiron® is believed to adhere to the bladder mucosal lining, thus maintaining or enhancing the urothelial permeability barrier.^8^ However, Elmiron® is a blood thinner and might increase the risk of bruising and bleeding. This treatment modality is listed as second‐line treatment by the American Urological Association, requires long‐term oral administration, and has low efficacy.^9^ Hyaluronic acid (HA) has been recently used to treat UCPPS via intravesical instillation. HA is a promising treatment, owing to its excellent biocompatibility and lack of biotoxicity.^10^ However, urination accelerates the loss of HA; thus, patients require monthly installations, which increases the discomfort and costs associated with the treatment.^11^

Cerium oxide (CeO_2_) nanoparticles (nanoceria, CNPs) display several promising benefits for the treatment of UCPPS, such as low cost, fewer adverse effects, and distinctive mechanisms of action in the management of chronic pain.^12^ CNPs demonstrate remarkable antioxidant properties by scavenging hydroxyl radicals, peroxynitrite, nitric oxide, and other free radicals and remaining active in tissues for prolonged periods via spontaneous redox switches.^13^ CNPs help in preventing or treating numerous acute and chronic conditions, including inactivation of human coronavirus,^14^ neuroprotection,^15^ anti‐diabetic properties,^16^ wound healing,^17^ and ovarian cancer.^18^ Hence, the requirement of CNPs for the prevention and treatment of chronic inflammation is rapidly increasing. Moreover, elucidating the role of serpin family B member 2 (SerpinB2), a protein implicated in the interaction between inflammation and cellular aging, is a priority.^19^ Mouse SerpinB2 mRNA is detected mainly in the urinary bladder, skin, thymus, bone marrow, spleen, and lung. SerpinB2 has been associated with asthma^20^ and chronic kidney disease.^21^ However, its potential involvement in the development of UCPPS has not been described.

Therefore, in this study, we aimed to investigate the in vivo preventative and therapeutic effects of CNPs in CYP‐induced chronic cystitis, and the molecular mechanism underlying SerpinB2 activity in UCPPS.

## 2 RESULTS AND DISCUSSION

### 2.1 Characterization of CNPs

**FIGURE 1:**
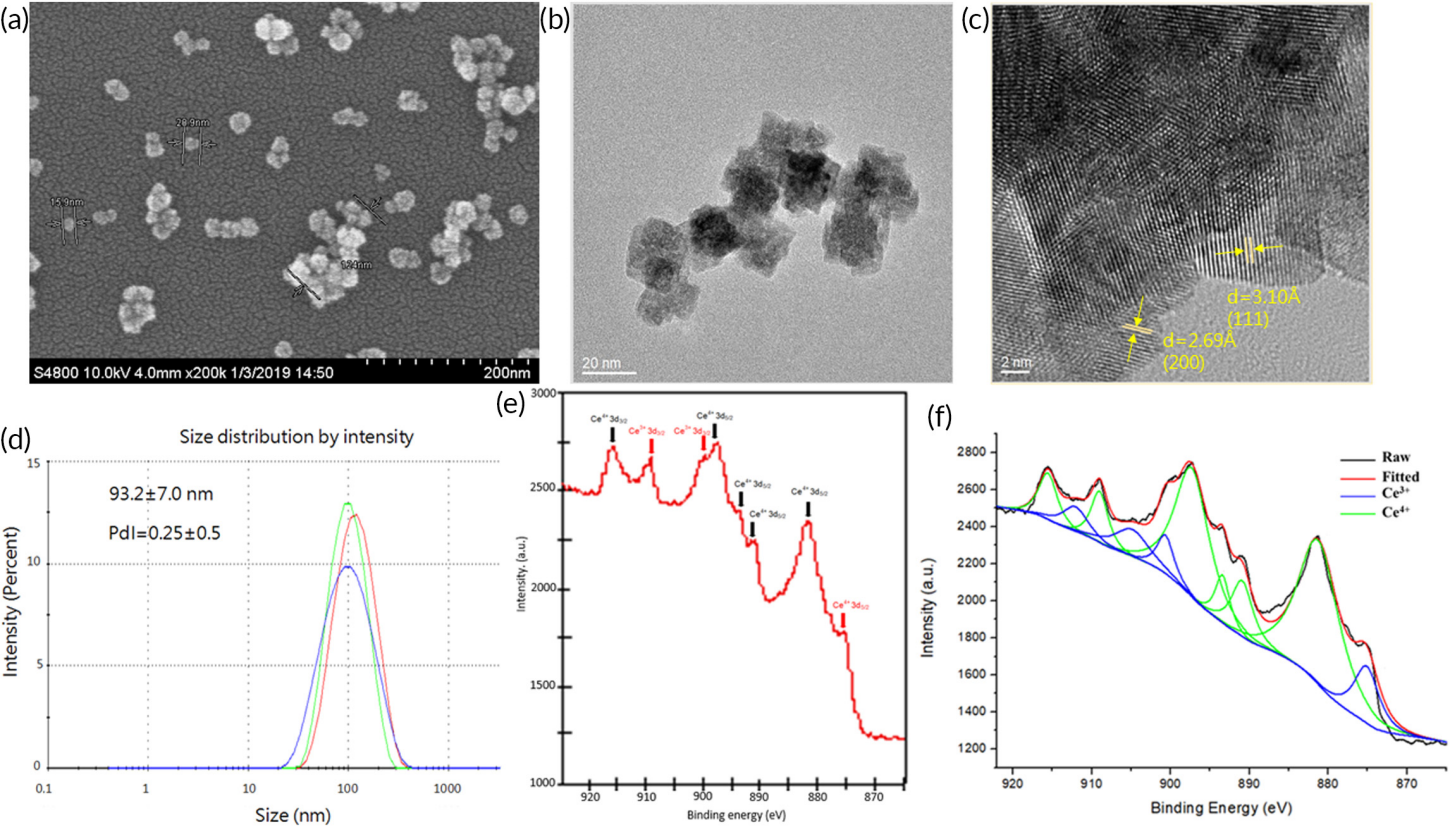
Characterization of cerium oxide nanoparticles (CNPs). (a) Scanning electron microscopy imaging of CNPs. CNPs were observed at 200,000× magnification (scale bar: 200 nm). (b) Transmission electron microscopy (TEM) imaging of CNPs at 150,000× magnification (scale bar: 20 nm). (c) TEM imaging of CNPs at 800,000× magnification (scale bar: 2 nm). (d) The hydrodynamic size distribution of CNP. (e) The X‐ray photoelectron spectroscopy (XPS) spectrum of CNP revealed the binding energy range of cerium. The arrows in red and black indicate the peaks of Ce^3+^ and Ce^4+^, respectively. (f) The fitted and de‐convoluted XPS spectrum of CNP

The mean CNP diameter (22.53 nm, *n* = 10; *n* is the number of particles measured) was measured using scanning electron microscopy (SEM) and Nano Measurer software and the CNP particles were cube/octahedron‐like in shape (Figure 1a). The larger nanoparticles may be due to the aggregation of the smaller ones, as the exposed surface was unstable, because the SEM and transmission electron microscopy (TEM) images indicated that the grain size of CNP was around 20 nm^10^ (Figure 1a,b). The high‐resolution TEM image of CNPs showed lattice fringes and *d* spacing related to the 111 and 200 crystalline planes, which matched standard cerium oxide (standard data JCPDS 34‐0394) (Figure 1c). The hydrodynamic size distribution and polydispersity index of CNPs in double‐distilled water were 93.2 nm and 0.25, respectively (Figure 1d). X‐ray photoelectron spectroscopy (XPS) showed chemical bonding peaks related to Ce^3+^ (red arrows in Figure 1e) and Ce^4+^ (black arrows in Figure 1e) at the corresponding binding energies. The peaks between 895 and 875 eV corresponded to the Ce 3d5/2 degraded level, whereas those between 910 and 895 eV corresponded to the Ce 3d3/2 degraded level. The deconvoluted peaks located at 875.100, 900.650, and 909.000 eV corresponded to the Ce^3+^ oxidation states. The deconvoluted peaks observed at 881.240, 890.900, 893.400, 897.300, and 915.550 eV could be ascribed to the Ce^4+^ state of the cerium ions (Figure 1f). A Ce^3+^/Ce^4+^ ratio of 1.49 was found in the CNPs by fitting and deconvolution of the XPS spectrum.

In the present study, we synthesized CNPs using a slight modification of the metal‐salt hydrolysis method.^22^ While the most abundant element in the rare‐earth family, cerium has two stable oxidation states, Ce^3+^ and Ce^4+^ (Figure 1e,f). The metal‐salt hydrolysis method of synthesizing CNPs at room temperature^22^ is more conducive to future laboratory production or the industrial mass production of this material. The coexistence of Ce^3+^ and Ce^4+^ results in self‐regenerative antioxidant ability and long‐term therapeutic potential.

#### 2.1.1 Effects of 4‐hydroperoxycyclophosphamide on T24 cell viability, the rescue of 4‐HC‐induced loss of T24 cells via CNP treatment, intracellular reactive oxygen species measurements, and expressions of pro‐inflammatory cytokines

**FIGURE 2:**
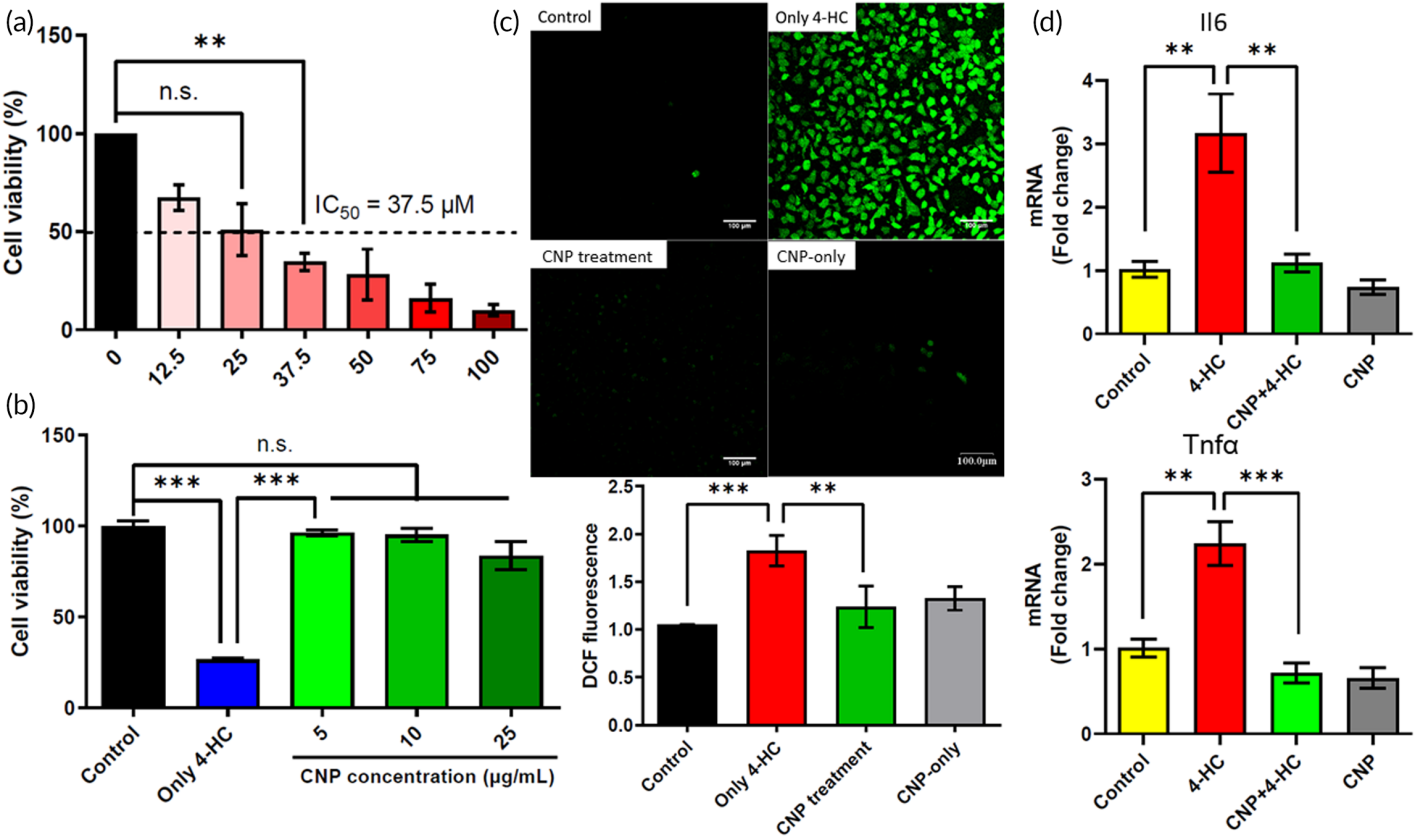
Effects of 4‐hydroperoxycyclophosphamide (4‐HC) on T24 human urothelial cell (BCRC no. 60062, Taiwan) viability, rescue of 4‐HC‐induced loss of T24 cells via CNP treatment, intracellular reactive oxygen species measurements, and expressions of pro‐inflammatory cytokines. (a) The T24 cell viability decreased following 4‐HC treatment, and intracellular reactive oxygen species generation was induced. T24 cells were treated with 4‐HC at concentrations of 0, 12.5, 25, 37.5, 50, 75, and 100 μM for 4 h. Cell viability was determined using a WST‐1 assay. At a 4‐HC concentration of 37.5 μM, the cell viability of T24 cells decreased to approximately 50% (*n* = 3). (b) CNP treatment prevented 4‐HC induced loss of T24 viability (*n* = 3). (c) Results of the 2′,7′‐dichlorofluorescin diacetate (DCFDA) assay of T24 cells. Fluorescence microscopy images of four cell groups: (1) control group, (2) 4‐HC group: 37.5 μM 4‐HC added for 4 h to induce cellular oxidative stress, (3) CNP treatment group: 5 μg/ml CNP applied for 24 h before 4 h of induction with 37.5 μM of 4‐HC, and (4) CNP‐only group. CNP was applied at a concentration of 5 μg/ml for 24 h. The amount of green fluorescence (dichlorofluorescin) was positively correlated with the intracellular ROS content. (d) Tests to confirm that CNP had an anti‐inflammatory effect on T24 cells treated with 4‐HC. 4‐HC group: 37.5 μM of 4‐HC added for 4 h to induce cellular oxidative stress. CNP + 4‐HC group: 5 μg/ml of CNP applied for 24 h before 4 h of induction with 37.5 μM of 4‐HC. CNP group: only 5 μg/ml of CNP applied for 24 h. *IL‐6* and *Tnfα* were significantly upregulated by 4‐HC; however, they were downregulated in the CNP + 4‐HC group. In the CNP groups, the cells did not exhibit excessive expression of inflammation‐related genes, confirming that the application of 5 μg/ml CNP for 24 h does not induce cell inflammation. The values are presented as mean ± standard deviation. Data were compared via one‐way analysis of variance with Tukey's post hoc tests. *p < 0.05, **p < 0.01, ***p < 0.001, *p* > 0.05: no significant difference (n.s.), *n* = 3. CNP, cerium oxide nanoparticles

Treatment with 37.5 μM of 4‐HC caused the cell viability rate to decrease to 50% (IC_50_). This concentration was set as the induction concentration for subsequent experiments (Figure 2a). Different concentrations of CNPs were applied before 4‐HC induction. CNP was well tolerated by T24 cells, as indicated by the absence of identifiable manifestations of toxicity. After a series of experiments, CNP (at a concentration of 5 μg/ml) effectively altered the reduction in cell viability induced by 4‐HC (Figure 2b). The 2′,7′‐dichlorofluorescin diacetate (DCFDA) assay confirmed that 4‐HC (at a concentration of 37.5 μM) could significantly induce reactive oxygen species (ROS) production in T24 cells. The amount of green fluorescence (dichlorofluorescin) positively correlated with the intracellular ROS content. To confirm the inhibitory effect of CNPs on the 4‐HC‐induced cellular oxidative stress response, T24 cells were cultured under four conditions: (1) control, (2) in the presence of 37.5 μM of 4‐HC for 4 h to induce cellular oxidative stress, (3) in the presence of 5 μg/ml of CNP for 24 h before 4 h of induction with 37.5 μM of 4‐HC, and (4) in the presence of 5 μg/ml of CNP for 24 h. The CNPs had an inhibitory effect on the 4‐HC‐induced cellular oxidative stress response (Figure 2c). The DCFDA assay revealed the capacity of CNPs to overcome oxidative stress caused by 4‐HC in vitro. In previous studies, CNPs also scavenged excessive ROS in acute ischemic conditions of critical limb ischemia, improved endothelial survival, and induced angiogenesis to revascularize an ischemic limb.^23^ Oxidative stress is often accompanied by pro‐inflammatory cytokine expression.^24^, ^25^ qPCR assays of *interleukin 6* (*Il6*) and *tumor necrosis factor α* (*Tnfα*) were performed in each group of cells. The four groups of cells were collected and subjected to RNA extraction for qPCR assays. The qPCR assay results are shown in Figure 2d. The qPCR assays of *Il6* and *Tnfα* revealed the capacity of CNPs to downregulate pro‐inflammatory cytokines, including IL‐6 and TNF‐α caused by 4‐HC in vitro. A previous study using osteoarthritis‐mimicking chondrocytes/macrophages co‐culture models found that the CNPs scavenged excessive ROS generated in the osteoarthritis joint in vivo and in vitro using the osteoarthritis‐mimicking chondrocytes/macrophages co‐culture models, where the immunomodulatory role of CNPs was shown to be the underlying molecular mechanisms.^26^

#### 2.1.2 Behavioral and histological characterization of CYP‐induced cystitis and the pretreatment effects of CNPs

**FIGURE 3:**
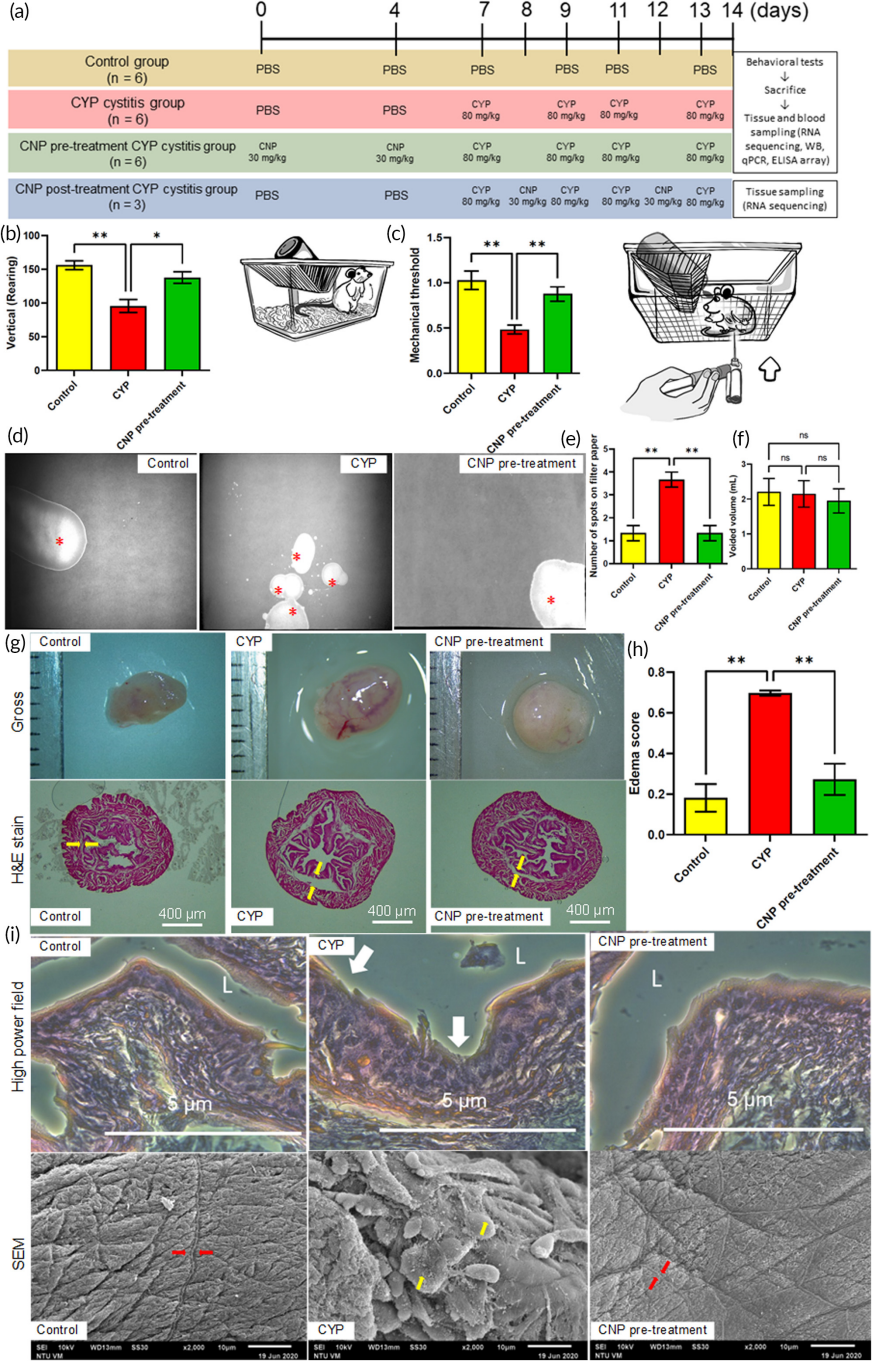
Behavioral and histological characterization of CYP‐induced cystitis and the pretreatment effects of CNPs. (a) Schedule of the animal experiments. The experiments were conducted on 8‐week‐old male ICR mice. All materials were administered via intraperitoneal injection. Rearing activity count and mechanical pain threshold of the control, CYP, and CNP pretreatment groups are shown in (b) and (c). The illustrations of rearing activity of the mouse and the test of mechanical pain threshold are shown in (b) and (c). (d) Representative voiding patterns in the control, CYP, and CNP pretreatment groups. Voiding spots are identified by *. Quantification of number of individual voiding spots and voided volume among the control, CYP, and CNP pretreatment groups are shown in (e) and (f). (g) Findings on gross examination and H&E staining. Urinary bladders of animals with CYP‐induced cystitis showing hyperemia and increased suburothelial thickness (between yellow arrows) compared with the control and CNP pretreatment groups. (h) Distributions of edema score. (i) High‐power field and scanning electron microscopy (SEM) images. These samples revealed the loss of urothelial integrity and an almost completely denuded urothelium (white arrows in high‐power field and red arrows in SEM) in the urinary bladders of animals with CYP‐induced cystitis, when compared with those in control and CNP pretreatment groups. Tight junctions were indicated between yellow arrows. CYP, cyclophosphamide; CNP, cerium oxide nanoparticles. The values are presented as mean ± standard error. Data were compared via one‐way analysis of variance with Tukey's post hoc tests. *p < 0.05, **p < 0.01. *p* > 0.05: no significant difference (n.s.), *n* = 3

Animal experimentation protocol is shown in Figure 3a. Animals with CYP‐induced cystitis showed decreased rearing activity and pain threshold. In contrast, an opposite trend was observed in these parameters in the CNP pretreatment group (*n* = 3 per group, *p < 0.05), as shown in Figure 3b,c. Animals with CYP‐induced cystitis exhibited an increased voiding frequency, which was alleviated in the CNP pretreatment group (*n* = 3 per group, *p < 0.05), as shown in Figure 3d,e.

At the macroscopic level, the urinary bladders of animals treated with CYP exhibited edema and hyperemia^27^ in comparison with the control group (Figure 3g). Furthermore, increased suburothelial thickness and the edema scores^28^ were observed among the CYP‐treated animals, compared to control group (Figure 3g,h).^29^ These samples were observed via scanning electron microscopy (SEM), and hematoxylin and eosin (H&E) staining revealed the loss of urothelial integrity and an almost completely denuded urothelium (white arrows in high‐power field and red arrows in SEM) in the urinary bladders of animals with CYP‐induced cystitis, when compared with those in the control and CNP pretreatment groups (Figure 3i). Tight junctions are indicated between yellow arrows (Figure 3i). Tight junction proteins, including occludin and zona occludens‐1 (ZO‐1) were downregulated after CYP induction, compared to their levels in control and CNP pretreatment groups (*n* = 3 in each group) (Figure S1).

The mechanical pain threshold and voiding spot assay revealed that CNP administration had an analgesic effect and reduced urinary frequency in the chronic CYP‐cystitis mouse model. Analysis of H&E staining and SEM of the bladder mucosa and urothelial cells of ICR mice revealed that CNP pretreatment had a protective effect on the bladder tissue barrier and helped maintain cellular and mucosal integrity. In recent studies, single topical instillation of the pilocarpine‐loaded nanoceria onto experimentally glaucomatous eyes mitigated disease progression for 7 days while treatment with commercial eye drops only provided a moderate treatment efficacy for 4 h.^30^ Based on existing data, the CNPs also have a long‐lasting potential (>7 days) to prevent the development of CYP‐induced UCPPS while a daily intake of the nonsteroid anti‐inflammatory drugs (NSAIDs) is required and the effects of NSAIDs on UCPPS are limited to the duration of therapy.^31^

#### 2.1.3 Cytokines and cytokine‐related gene expression in the bladder and serum cytokine levels

**FIGURE 4:**
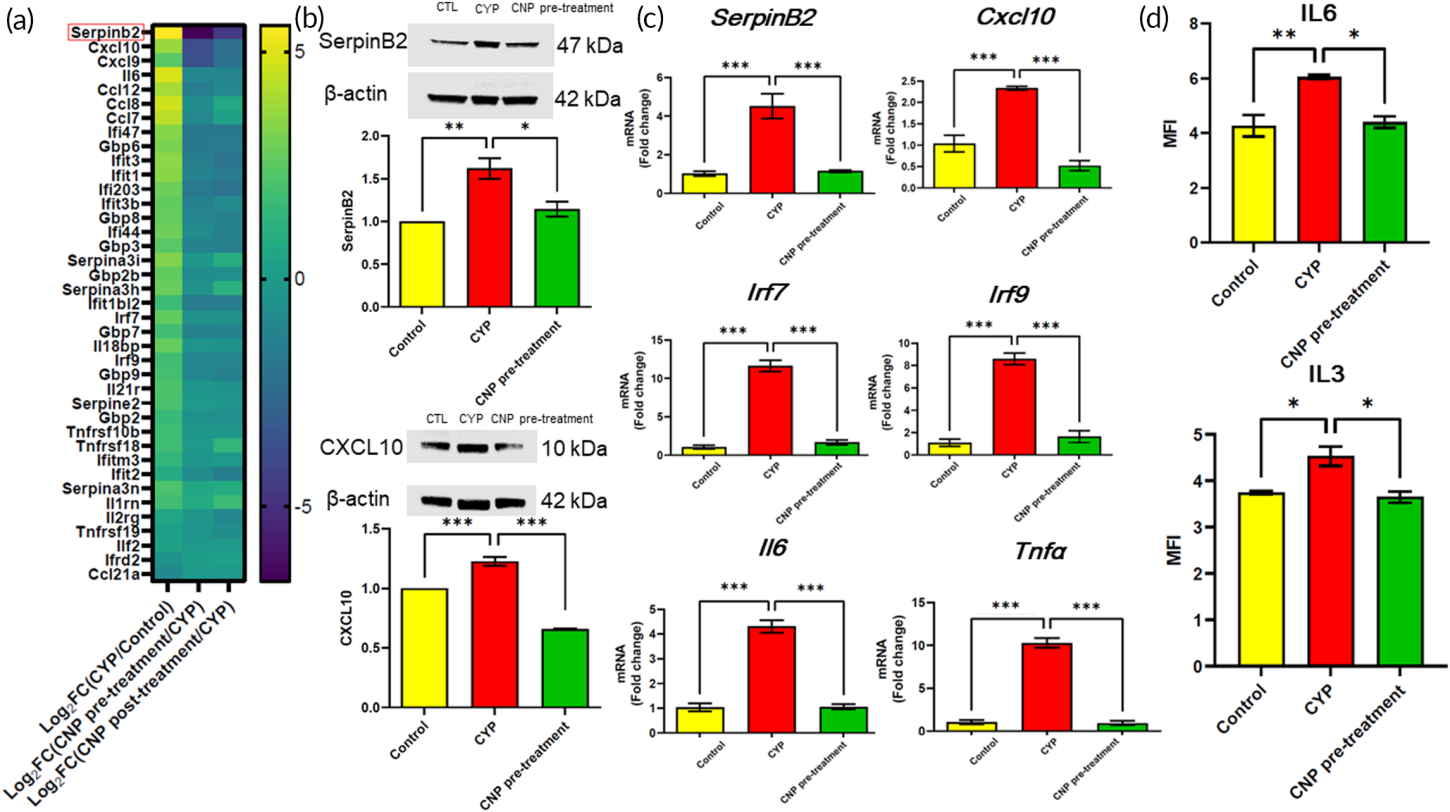
Cytokines and cytokine‐related gene expression in the bladder and serum cytokine levels. (a) Heat map of the differentially expressed genes associated with inflammation in the CYP versus control groups, the CNP pretreatment versus CYP groups, and the CNP posttreatment versus CYP groups. (b) Relative amounts of SerpinB2 and CXCL10 in the bladders of mice in the control, CYP, and CNP pretreatment groups. (c) Results of CNPs on the expression of cytokine‐encoding and cytokine‐related genes. (d) Results of differential serum pro‐inflammatory cytokines among the control, CYP, and CNP pretreatment groups. *p < 0.05. CYP, cyclophosphamide; CNP, cerium oxide nanoparticle; MFI, mean fluorescent intensity. The values are presented as mean ± standard error. Data were compared via one‐way analysis of variance with Tukey's post hoc tests. *p < 0.05, **p < 0.01, ***p < 0.001. *p* > 0.05: no significant difference (n.s.), *n* = 3

After RNA sequencing, cytokine and cytokine‐related gene expressions exhibited in the urinary bladders of the animals treated with CYP were substantially upregulated compared to those in control and CNP pretreatment groups. The comparison of the heatmap between the columns of log_2_FC(CNP pretreatment/CYP) and log_2_FC(CNP posttreatment/CYP) revealed more reduction of cytokine‐related gene expressions in the CNP pretreatment groups than that in posttreatment groups (Figure 4a). Hence, in this study, we adopted the protocol of CNP pretreatment as the preventative regimen. Enrichment analysis of Gene Ontology (GO) response to cytokines in the biological process (BP) category was shown in Figure S2. The *SerpinB2* gene was the most upregulated among the cytokine and cytokine‐related genes in the CYP groups when compared with that in the control groups, and the most downregulated among the cytokine and cytokine‐related genes in the CNP pretreatment groups when compared with that in the CYP groups, as shown in Figure 4a. The less effective immunomodulatory effects of CNP protocol posttreatment may be due to the disruption of urothelial integrity (Figure 3i) and downregulation of tight junction proteins after CYP induction (Figure S1).^32^, ^33^

Following Western blotting, increased levels of SerpinB2, CXCL10 (Figure 4b), and HO‐1 (Figure S3) were observed in the urinary bladders of animals treated with CYP in comparison with those observed in control and CNP pretreatment groups. The results of the quantitative polymerase chain reaction (qPCR) assay showed that the urinary bladders of the animals treated with CYP had increased expressions of *Irf7*, *Irf9*, *Cxcl10*, *Ifit1*, *Serpinb2*, *Il6*, and *Tnfα*, shown in Figure 4c, and *Hmox1*/*HO‐1* ^33^ (Figure S3) compared with those of animals in the control group. The increased HO‐1 expression indicated oxidative stress in CYP‐cystitis. The downregulation of HO‐1 in vivo confirmed its potential of long‐acting ability to inhibit oxidative stress.^34^

IL‐6 is one of the main inflammatory cytokines, produced by macrophages, and stimulated by IL‐1 beta. IL‐3, produced by macrophages and stromal cells, may play a role in chronic inflammatory conditions and is a multicolony stimulating factor for granulocytes, macrophages, erythroid cells, and mast cells.^35^ Serum levels of IL‐6 and IL‐3 were higher in the CYP cystitis group than in the control and CNP pretreatment groups (*n* = 3 in each group) using a Quantibody Multiplex ELISA array (RayBiotech) (Figure 4d). The results revealed that CNP successfully decreased pelvic pain and bladder inflammation among mice exposed to fourfold serial low‐dose CYP induction. CNP diminished CYP‐induced oxidative stress and inflammation in both in vivo and in vitro UCPPS models and signified the immunomodulatory potential of SerpinB2. With progress through the proposed line of research, UCPPS may be considered a new indication for the application of CNPs.

The clinical characteristics of UCPPS include familial clustering and elevated recurrence risk^36^; therefore, CNP pretreatment may be considered to prevent the occurrence of familial clustering and the frequent recurrence of UCPPS. Currently, the concept of multimodal analgesia is broadly credited in the management of visceral pain.^37^ Adverse events related to most affordable nonopioid analgesics (e.g., nonselective nonsteroid anti‐inflammatory drugs, cyclooxygenase‐2 inhibitors, alpha‐2 agonists, ketamine, and local anesthetics) increase the risk of bleeding, peptic ulcer disease, and kidney injury.^38^ Opioid‐related adverse events, including nausea, vomiting, bladder dysfunction, alimentary stoppage, itchiness, visual hallucinations, sedation, respiratory depression, lasting physical dependence, and addiction may also occur. Even the limited usage of strong opioid analgesics can provoke hyperesthesia due to opioid‐induced hyperalgesia.^39^, ^40^ Moreover, opioid analgesic treatment aimed at relieving chronic pain may provoke opioid‐induced hyperalgesia.^39^, ^40^ The combined use of CNPs could diminish the possibility of respiratory depression, nausea and vomiting, alimentary stoppage, and bladder dysfunction under postsurgical conditions. It could provide an improved margin of safety for high‐risk patients (e.g., elderly, obese, and patients with multiple comorbidities).^41^ In this study, we found that the use of CNPs represents a new strategy for analgesia treatment. Under the concept of multimodal analgesia, immunomodulation by CNPs potentially enhances effects such as analgesia treatment and reduces the incidence of adverse events.

#### 2.1.4 Human bioinformatics analysis: SerpinB2 siRNA repressed 4‐HC‐induced elevated inflammatory cytokine levels in T24 cells

**FIGURE 5:**
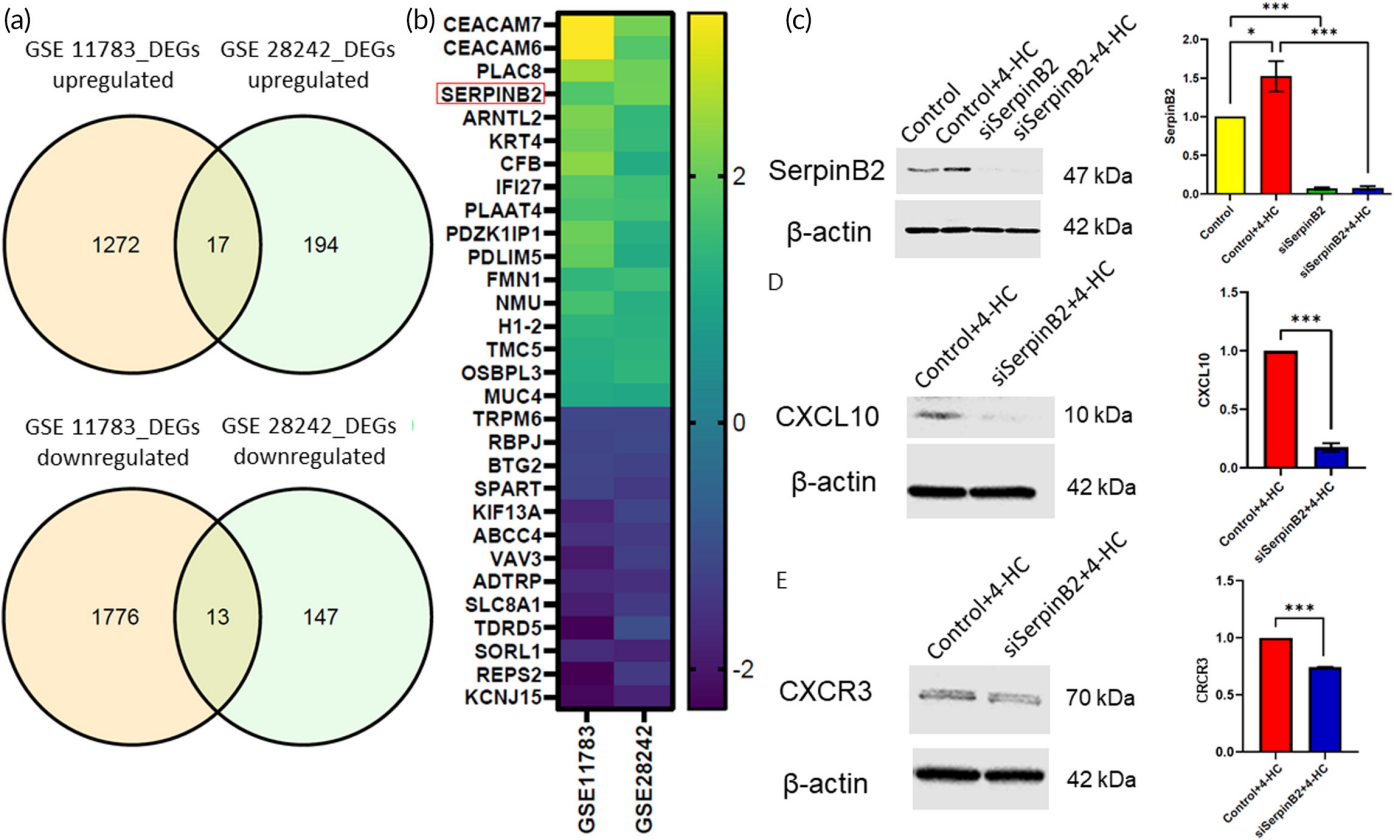
Bioinformatics analysis: SerpinB2 siRNA repressed 4‐hydroperoxycyclophosphamide (4‐HC)‐induced elevated inflammatory cytokine levels in T24 cells. (a) There were 30 genes in the non‐Hunner type cystitis group that collaborated between GSE11783^42^ and GSE28242.^43^ (b) Seventeen of the genes were upregulated, including *SerpinB2*, while 13 genes were downregulated in the non‐Hunner type cystitis group in comparison with the normal control group as illustrated in the heat map. (c) The T24 cells in the control and 4‐HC groups and the transfected T24 cells with SerpinB2 knockdown treated with and without 4‐HC were assessed via Western blotting of SerpinB2. (d) The expression of CXCL10 was significantly reduced by SerpinB2 small interfering RNA (siSerpinB2) in T24 cells treated with 4‐HC. (e) The expression of CXCR3 was significantly reduced by siSerpinB2 in T24 cells treated with 4‐HC. The values are presented as mean ± standard error. Data were compared via one‐way analysis of variance with Tukey's post hoc tests. *p < 0.05, **p < 0.01, ***p < 0.001, *p* > 0.05: no significant difference (n.s.), *n* = 3

The expression of UCPPS‐related genes in the urinary bladder and urine of human subjects with UCPPS was substantially increased compared with that of subjects in the control group. The results revealed that 30 genes in the non‐Hunner type cystitis group were collaborated between GSE11783^42^ and GSE28242,^43^ in which 17 genes were upregulated, including *SerpinB2*, and 13 genes were downregulated in the non‐Hunner type cystitis group in contrast to the normal control group (Figure 5a,b). Enrichment analysis of GO terms of upregulated BP was shown in Figure S4. The T24 cells in the control and 4‐hydroperoxycyclophosphamide (4‐HC) groups and the transfected T24 cells with SerpinB2 knockdown treated with and without 4‐HC were assessed via Western blotting of SerpinB2. The siRNA targeting of SerpinB2 successfully downregulated SerpinB2 expression (Figure 5c). We investigated chemokine expression in urothelial cells and found a 0.18‐fold decrease (*n* = 3 per group, ***p < 0.001, unpaired two‐tailed *t*‐tests) and a 0.74‐fold reduction (*n* = 3 per group, ***p < 0.001, unpaired two‐tailed *t*‐tests) in the protein excretion of CXCL10 and CXCR3 from urothelial T24 cell SerpinB2 knockdown cultures, respectively (Figure 5d,e).

CXCL9, CXCL10, and CXCL11 are chemotactic in white blood cells and trigger T lymphocytes. A previous study revealed a significant elevation in the level of CXCL10 in contrast to CXCL9 and CXCL11 in experimental autoimmune cystitis.^7^ A blockade of CXCL10 affects other ligands at the cellular level, leading to a moderate systemic depletion of CXCL9 and CXCL11, while ameliorating the severity of cystitis. UCPPS is a disease of the urothelium,^44^ with urothelial dysfunction as an initial step in the potential etiologic cascade.^45^ Bladders from patients with UCPPS have increased the expression of CXCR3 in the urothelium.^46^ Furthermore, the expression of SerpinB2 was upregulated via 4‐HC induction in T24 human urothelial cells (Figure 5c). The immunomodulatory potential demonstrated that the knockdown of SerpinB2 resulted in the downregulation of the pro‐inflammatory chemokine CXCL10 and chemokine receptor CXCR3^47^ in human urothelial T24 cells. The downregulation of CXCL10 in the bladder tissue may play an immunomodulatory role in the management of UCPPS, and the downregulation of SerpinB2 may be also associated with decreased CXCL10 expression in the CYP cystitis mouse model pretreated with CNP.

Non‐Hunner type cystitis was considered a common finding, accounting for 90% of cases with poor overall treatment outcomes.^48^ SerpinB2 was upregulated in non‐Hunner type lesions in both humans and the CYP cystitis mouse model. SerpinB2, also known as plasminogen activator inhibitor 2 type A, expression was substantially downregulated in mice pretreated with CNP. SerpinB2 is the hub gene in human UCPPS pathogenesis based on protein–protein interactions (Figure S5). SerpinB2 affects the ordinary fibrinolytic process in thrombus resolution.^49^ Dysregulated SerpinB2 expression is associated with pre‐eclampsia, lupus, asthma, scleroderma, periodontitis,^50^ and UCPPS in human and mouse models. SerpinB2 blockade reduces the expressions of the chemokine receptor CXCR3 and proinflammatory chemokine CXCL10 in T24 human urothelial cells treated with 4‐HC.

#### 2.1.5 Study limitations

Our study had certain limitations. First, we only evaluated animals pretreated with CNP. It is feasible that the therapeutic effect of CNP treatment on the regulation of bladder inflammation after CYP‐induced cystitis could have been investigated with CNP treatment after serial injections of CYP. The less effective immunomodulatory effects of CNP protocol posttreatment may be due to the disruption of urothelial integrity (Figure 3i) and delays to start of treatment that are unrealistic in the clinic. On the other hand, this CNP pretreatment preventative regimen demonstrated long‐lasting protective potential in mice exposed to induction with a fourfold serial low‐dose of CYP. Thus, the only naturally occurring chronic model (feline interstitial cystitis), not involving noxious stimulation of the bladder, may be considered in the investigation of CNP posttreatment therapeutic effects.^51^ Second, the underlying mechanism by which *SerpinB2* knockdown leads to CXCL10 downregulation remains unknown. SerpinB2 knockdown reduces the expression of the CXCR3 and may lead to CXCL10 downregulation following CXCL10:CXCR3 axis^47^ in T24 human urothelial cells. Further studies are required to clarify the signaling pathways underlying the role of *SerpinB2* and the involvement of proinflammatory chemokines such as CXCL10 in UCPPS in human and mouse models. Finally, the clinical applicability of small animal models for an overactive bladder is still questioned, as urinary frequency, voided volume, and bladder wall stiffness vary from what is observed clinically. However, the current mouse cystitis model constitutes fundamental work for future research evaluating the effects of CNP treatment on UCPPS pathogenesis using larger animal models and even among human patients.

### 2.2 Materials and Methods

#### 2.2.1 Synthesis of cerium oxide nanoparticles

CNPs were synthesized using a slight modification of a previously described method.^22^ Briefly, equal volumes of 0.0375 mol/L Ce(NO_3_)_3_·6H_2_O (99.5%; Alfa Aesar, Ward Hill, MA, USA) and 0.5 mol/L hexamethylenetetramine (99.9%, Alfa Aesar) solutions were mixed and stirred in a temperature‐controlled environment at approximately 24 ± 2°C for 24 h. Then, the solution was centrifuged at 9000 rpm for 30 min to obtain CNP sediments. The sediments were washed twice with deionized water and ethanol (95%; Alfa Aesar) for the SEM measurement samples. The resulting CNPs were resuspended in PBS solution and sterilized in an autoclave at 121°C for 15 min for in vitro and in vivo studies.

#### 2.2.2 Morphology of CNPs

The morphology and particle size of the CNPs were evaluated via SEM (Hitachi S‐4800 Field Emission Scanning Electron Microscope [Hitachi Ltd., Japan] with the energy‐dispersive X‐ray spectrometer QUANTAX Annular XFlash® QUAD FQ5060 [Bruker, Middlesex, NJ, USA]) at an operating voltage of 10 kV. The specimens were mounted onto an adhesive copper stub and then gold sputtered. The SEM images (*n* = 10; *n* is the number of particles measured) of the CNPs were analyzed using the Nano Measurer 1.2 software to obtain the average size of the CNPs. For the SEM morphological analysis, the attached accessory energy‐dispersive x‐ray spectrometer (QUANTAX Annular XFlash®, QUAD FQ5060) was used to analyze the elemental composition of the samples. The SEM images of the CNPs were observed at ×200,000 magnification (scale bars represent 200 nm) (Figure 1a). The methods and preparation of measurement samples of TEM, hydrodynamic size, x‐ray diffraction, specific surface area, and surface characterization of CNPs are provided in the supplementary materials S1.

#### 2.2.3 Human urothelial cell culture and UCPPS model establishment in vitro

T24 human urothelial cells (BCRC no. 60062, Taiwan) were cultured in 90% McCoy's 5A Medium (Sigma‐Aldrich) with 1.5 mM l‐glutamine, 10% (v/v) fetal bovine serum, and 1% (v/v) penicillin/streptomycin. The cells were maintained at 37°C in a humidified incubator containing 5% CO_2_.^52^ In the present study, 4‐HC was applied in vitro to induce T24 cells to produce oxidative stress and apoptosis. In an aqueous solution, 4‐HC can rapidly hydrolyze to 4‐hydroxycyclophosphamide, the same intermediate product resulting from the microsomal activation of CYP,^53^ and therefore, 4‐HC was used instead of CYP because of the need of CYP for microsomal activation. To assess the effects of CNP treatment in the UCPPS model established in vitro, T24 cells were subdivided into four groups: control, 4‐HC, CNP pretreatment, and only CNP groups. The methods used in the water‐soluble tetrazolium salt‐1 and DCFDA cellular ROS detection assays are provided in the supplementary materials S1.

#### 2.2.4 Experimental animals: ICR mice with cyclophosphamide‐induced cystitis

The study protocol was approved by the Institutional Animal Ethics Committee of our university (IACUC approval no. 110065) as per the National Research Council's Guide for the Care and Use of Laboratory Animals. The study was carried out using male ICR mice (age, 6–8 weeks; weight, 25–30 g). The animals were housed in a climate‐controlled environment at approximately 24 ± 2°C with a 12/12‐h light/dark cycle and relative humidity of 55% ± 15% during the entire experimental period. They were observed for any signs of disease and maintained under standard conditions until the end of the experiment.

#### 2.2.5 Experimental group design

The experimental animals were subdivided into three groups: control, CYP, CNP pretreatment groups, and CNP posttreatment groups. The experimental animals were euthanized on Day 14 after the behavioral experiments were conducted. The control animals were intraperitoneally injected with sterile phosphate buffered saline (PBS) six times over 14 days. Chronic bladder inflammation was induced using CYP. In the CYP group, 8 mg/ml of CYP (C0768; Sigma‐Aldrich, St. Louis, MO, USA) was diluted in sterile PBS, and 80 mg/kg of the final solution was administered intraperitoneally four times, which is the most commonly used mouse model to study UCPPS according to Multidisciplinary Approach to the Study of Chronic Pelvic Pain (MAPP) Research Network.^3^ The injections were administered on Days 7, 9, 11, and 13. To ensure that each group of mice received the same number of injections, mice in the CYP group were intraperitoneally injected with sterile PBS on Days 0 and 4. CNP (6 mg/ml) was dispersed in sterile PBS, and the mice in the CNP pretreatment group were injected with the final solution at a concentration of 30 mg/kg on Days 0 and 4 and then received CYP as indicated for the CYP group. The mice in the CNP posttreatment group were injected with the final solution at a concentration of 30 mg/kg on Days 8 and 12 during CYP administration to investigate the RNA sequencing of the urinary bladder. Before each injection, the mice were weighed to calculate the volume of intraperitoneal injections of PBS, CYP, and CNP solutions. The design of the animal experiments is illustrated in Figure 3a.

#### 2.2.6 Mechanical pain threshold testing

The mechanical pain threshold was evaluated using Semmes–Weinstein monofilaments (Ugo Basile, Comerio, Italy).^54^ The mice were assessed in isolated cages with a stainless‐steel wire grid floor. The stimulation was restricted to the lower abdominal area in the common region of the bladder and was performed in separate areas inside this region to prevent desensitization. Three behavioral patterns were recognized as positive reactions to monofilament stimulation: (1) jumping, (2) immediate licking or scratching of the area of monofilament stimulation, or (3) sharp abdominal retraction. Each monofilament was implemented for 1–2 s with an interstimulus interval of 5 s. The “up and down” method was utilized to identify the 50% threshold (T50) and six successive attempts with distinct monofilaments were required based on the estimation of the most reliable threshold.^55^ T50 was determined using the equation *T*50 = *Xf* + *kd* (where *Xf* represents the last monofilament used, *k* represents the coefficient determined from Dixon's table, and *d* represents the mean monofilament interval). A description of the rearing activity and voiding spot assays are provided in the supplementary materials S1.

#### 2.2.7 Histology

The urinary bladder of each mouse was observed macroscopically. The entire bladder of each mouse was frozen in liquid nitrogen immediately after harvesting (*n* = 3 in each group). To evaluate whole morphologic alterations, vesical cross sections (16‐μm thick) were stained using a standard H&E protocol, including 4 min in a Harris modified hematoxylin solution and 1 min in an Eosin Y alcoholic solution (Sigma‐Aldrich).

Each bladder was sliced by separating the dome of the bladder such that the inner space could be observed clearly. Eight slides (all for H&E staining) with eight sections on each slide were collected. This sectioning constituted the thickest midsection of the bladder. A previous study showed that at the microscopic level, the urinary bladders of animals subjected to CYP exhibit increased suburothelial thickness compared with the control group based on the edema score.^28^ For the SEM, samples were fixed in 2.5% phosphate buffered glutaraldehyde (0.1 M, pH 7.4) for 24 h, postfixed with 1% osmium tetroxide for 1 h, desiccated in a graded alcohol series, placed in amyl acetate, dehydrated with liquid carbon dioxide (CO_2_) under pressure with a critical point dryer, and covered with gold particles.

#### 2.2.8 Quantitative real‐time PCR assay

The bladders of the mice were snap‐frozen on CO_2_ (*n* = 3 in each group). Total RNA was extracted from the bladders and cells using a TRIzol reagent (Invitrogen, Carlsbad, CA, USA). Glyceraldehyde 3‐phosphate dehydrogenase was used as an endogenous control. cDNA was synthesized using the SensiFAST cDNA Synthesis kit (Bioline Ltd., London, UK) with random hexamer and polymer (dT) primers. PCR assays were performed using a qPCR system (StepOne™ Software version 2.2; Thermo Fisher Scientific, Waltham, MA, USA).^56^ Each sample was evaluated in triplicate for both the target gene and endogenous control. The mean cycle threshold (Ct) values of the triplicate samples were used for additional analyses. The Ct value for the target gene obtained from each sample was normalized to that of α‐tubulin, an internal reference gene, and transformed to relative gene expression values using the 2^−ΔΔCt^ method. The primer sequences are listed in Table S1, and the methods of RNA sequencing are provided in the Supplementary Methods S1.

#### 2.2.9 Western blot analysis of heme oxygenase 1, CXCL10, SERPINB2, occluding, and ZO‐1 (zonula occludens‐1) expressions

The bladder tissue and cell expressions of HO‐1, CXCL10, and the SerpinB2 proteins, the bladder tissue expressions of occludin and ZO‐1 proteins, and the cell expressions of the CXCR3 protein were analyzed by Western blotting. The primary antibodies, anti‐HO‐1 (#ab13248; Abcam, Cambridge, MA, USA), anti‐CXCL10 (#ab9938; Abcam), anti‐SerpinB2 (#ab269275; Abcam), anti‐Occludin (27260‐1‐AP; Proteintech, Rosemont, USA), anti‐ZO‐1 (21773‐1‐AP; Proteintech), anti‐CXCR3 (26756‐1‐AP; Proteintech), and anti‐β‐actin (#26276; GeneTex, Irvine, CA, USA) were incubated overnight at a 1:7000 dilution, at a temperature of 4°C. Next, the membranes were washed with washing buffer three times for 30 min, followed by incubation with a 1:6000 dilution of secondary anti‐rabbit (#213110–01; GeneTex) or anti‐mouse (#213110–01; GeneTex) IgG antibody at 37°C for 1 h. Finally, the membranes were rinsed with Immobilon Western Chemiluminescent Horseradish Peroxidase substrate (WBKLS0500; Millipore, Billerica, MA, USA), and protein bands were analyzed using a Hansor Luminescence Image System (model: M3‐8068; Hansor, Taichung, Taiwan).^57^

#### 2.2.10 Small interfering RNA transfection

To knock down SerpinB2 expression, T24 cells were transfected with siRNA against SerpinB2 (TOOLS Biotechnology Co., Taiwan) using Lipofectamine 2000 (Invitrogen Corporation, Carlsbad, CA, USA) according to the manufacturers' instructions.^58^ The sequences of SerpinB2 siRNA and the negative control are shown in Table S1.

T24 cells were seeded into a 96‐well plate at a density of 10^4^ cells/well and incubated for 24 h until full adhesion was achieved. The siRNA was initially transfected. Next, 37.5 μM of 4‐HC was added to the medium and incubated for 4 h.

#### 2.2.11 Statistical analysis

Data were collected from three separate experiments and expressed as mean ± standard deviation or the mean ± square error of the mean. The normality of distribution of the data was evaluated using the Kolmogorov–Smirnov test. Differences between two groups were compared using unpaired two‐tailed *t*‐tests or one‐way analysis of variance, combined with Tukey's multiple comparison tests for post hoc comparisons. We performed power analyses using G*Power version 3 software (Universität Düsseldorf, Düsseldorf, Germany)^59^ with one‐way analysis of variance, and the two‐sided significance level was set at *α* = 0.05. The effect size was evaluated in accordance with previous studies of the mouse bladder pain syndrome model.^60^ Using nine animals, the statistical power was calculated at *β* > 0.95. The statistical analyses were performed using SPSS version 20 (IBM Corp., Armonk, NY, USA) and GraphPad Prism version 5 (GraphPad Software, La Jolla, CA, USA). Analysis items with two‐tailed *p* values <0.05 were considered significant.

## 3 CONCLUSIONS

To the best of our knowledge, this is the first in vitro and in vivo study to investigate the therapeutic potential of CNPs in UCPPS. Our study showed that CNPs are well tolerated, without inducing toxicity in T24 cells and no serious adverse events were observed in vivo mice. Further, CNP usage in a multimodal analgesic strategy may provide analgesic effects without serious risk of side effects, warranting further exploration. The ROS scavenging and downregulation of CXCL10 in the bladder tissue may play an immunomodulatory role in the management of UCPPS, and the downregulation of SerpinB2 may be also associated with decreased CXCL10 expression in the CYP cystitis mouse model pretreated with CNP. Further work is needed to explore the duration of CNP pretreatment for protection against UCPPS development. Our work supports CNP administration as a potential preventative strategy for patients at higher risk for UCPPS.

## AUTHOR CONTRIBUTIONS

**Wei‐Chih Lien:** Conceptualization (equal); data curation (equal); formal analysis (equal); funding acquisition (equal); investigation (equal); methodology (equal); project administration (equal); resources (equal); validation (equal); visualization (equal); writing – original draft (equal); writing – review and editing (equal). **Xin‐Ran Zhou:** Conceptualization (equal); data curation (equal); formal analysis (equal); methodology (equal); validation (equal); visualization (equal); writing – original draft (equal); writing – review and editing (equal). **Ya‐Jyun Liang:** Conceptualization (equal); data curation (equal); formal analysis (equal); investigation (equal); methodology (equal); validation (equal); visualization (equal). **Congo Tak‐Shing Ching:** Conceptualization (equal); formal analysis (equal); methodology (equal); resources (equal); writing – review and editing (equal). **Chia‐Yih Wang:** Conceptualization (equal); formal analysis (equal); methodology (equal); resources (equal). **Fu‐I Lu:** Formal analysis (equal); investigation (equal); methodology (equal); resources (equal). **Huei‐Cih Chang:** Data curation (equal); formal analysis (equal); investigation (equal); validation (equal). **Feng**‐**Huei Lin:** Conceptualization (equal); formal analysis (equal); funding acquisition (equal); investigation (equal); methodology (equal); project administration (equal); resources (equal); software (equal); supervision (equal); validation (equal); writing – original draft (equal); writing – review and editing (equal). **Hui‐Min David Wang:** Conceptualization (equal); formal analysis (equal); funding acquisition (equal); investigation (equal); methodology (equal); project administration (equal); resources (equal); software (equal); supervision (equal); validation (equal); writing – original draft (equal); writing – review and editing (equal).

## FUNDING INFORMATION

This study was supported by the Ministry of Science and Technology, Taiwan (grant numbers MOST110‐2314‐B‐006‐096 to Wei‐Chih Lien; MOST110‐2221‐E‐005‐010 to Hui‐Min David Wang); the National Cheng Kung University Hospital, Taiwan (grant numbers NCKUH‐10909013, NCKUH‐11004013, and NCKUH‐11102065 to Wei‐Chih Lien); the ENABLE project of the National Chung Hsing University (grant number ENABLE:110ST001G); and Kasetsart University Joint Research Project (grant number: 108RA129A). The funders played no role in the study design, data collection and analysis, interpretation of data, or writing of the manuscript.

## CONFLICT OF INTEREST

The authors have no conflicts of interest to declare.

### PEER REVIEW

The peer review history for this article is available at https://publons.com/publon/10.1002/btm2.10346.

## Supporting information


**Appendix S1 Supplementary Text.** Supplementary Methods
**Supplementary Table S1**. List of primers for qPCR, SerpinB2 siRNA, and negative controls
**Supplementary Figure S1**. Expression of tight junction proteins in the bladder. (a) Relative amounts of occludin in the bladders of mice in the control, CYP, and CNP pretreatment groups. (b) Relative amounts of zonula occludens‐1 (ZO‐1) in the bladders of mice in the control, CYP, and CNP pretreatment groups. The values are presented as mean ± standard error. Data were compared using one‐way analysis of variance with Tukey's post hoc tests. *p < 0.05, **p < 0.01, ***p < 0.001, *n* = 3.
**Supplementary Figure S2**. Enrichment analysis of Gene Ontology (GO) terms of response to cytokines in the biological process (BP) category in the CYP versus control groups and the CYP versus CNP pretreatment groups in the urinary bladders of animals with CYP‐induced cystitis. Adjusted *p* value: Red < purple < blue.
**Supplementary Figure S3**. Expression of heme oxygenase 1 (HO‐1) in control, cyclophosphamide (CYP), and cerium oxide nanoparticles (CNP) pretreatment groups utilizing quantitative real‐time polymerase chain reaction (qPCR) assays (a) and Western blotting (b).
**Supplementary Figure S4**. Enrichment analysis of Gene Ontology (GO) terms of upregulated biological process (BP) in the non‐Hunner type cystitis group in comparison with the normal standard control group that collaborated between the human datasets of GSE11783^40^ and GSE28242.^41^ Adjusted *p* value: Red < purple < blue.
**Supplementary Figure S5**. Protein–protein interaction networks of the 30 differentially expressed genes in the non‐Hunner‐type cystitis group that collaborated between the datasets of GSE11783^42^ and GSE28242.^43^ Red arrows indicate the location of SerpinB2. Network nodes represent proteins. Edges indicate protein–protein associations. The color of the ring indicates expression level (red, upregulation; green, downregulation).

## Data Availability

GSE11783 and GSE28242 data were obtained from the NCBI GEO database (https://www.ncbi.nlm.nih.gov/geo/query/acc.cgi?acc=GSE11783 and https://www.ncbi.nlm.nih.gov/geo/query/acc.cgi?acc=GSE28242, respectively). All relevant data assessed in this study are available from the authors.
