# Supplementary material for: Therapeutic potential of nanoceria pretreatment in preventing the development of urological chronic pelvic pain syndrome: Immunomodulation via reactive oxygen species scavenging and SerpinB2 downregulation
Source: Bioeng Transl Med. 2022 Jun 13;8(1):e10346. doi: 10.1002/btm2.10346 (PMC9842028; doi:10.1002/btm2.10346)
Supplement: Supplementary file 1 — Appendix S1 Supplementary Text. Supplementary Methods Supplementary Table S1. List of primers for qPCR, SerpinB2 siRNA, and negative controls Supplementary Figure S1. Expression of tight junction proteins in the bladder. (a) Relative amounts of occludin in the bladders of mice in the control, CYP, and CNP pretreatment groups. (b) Relative amounts of zonula occludens‐1 (ZO‐1) in the bladders of mice in the control, CYP, and CNP pretreatment groups. The values are presented as mean ± standard error. Data were compared using one‐way analysis of variance with Tukey's post hoc tests. *p < 0.05, **p < 0.01, ***p < 0.001, n = 3. Supplementary Figure S2. Enrichment analysis of Gene Ontology (GO) terms of response to cytokines in the biological process (BP) category in the CYP versus control groups and the CYP versus CNP pretreatment groups in the urinary bladders of animals with CYP‐induced cystitis. Adjusted p value: Red < purple < blue. Supplementary Figure S3. Expression of heme oxygenase 1 (HO‐1) in control, cyclophosphamide (CYP), and cerium oxide nanoparticles (CNP) pretreatment groups utilizing quantitative real‐time polymerase chain reaction (qPCR) assays (a) and Western blotting (b). Supplementary Figure S4. Enrichment analysis of Gene Ontology (GO) terms of upregulated biological process (BP) in the non‐Hunner type cystitis group in comparison with the normal standard control group that collaborated between the human datasets of GSE1178340 and GSE28242.41 Adjusted p value: Red < purple < blue. Supplementary Figure S5. Protein–protein interaction networks of the 30 differentially expressed genes in the non‐Hunner‐type cystitis group that collaborated between the datasets of GSE1178342 and GSE28242.43 Red arrows indicate the location of SerpinB2. Network nodes represent proteins. Edges indicate protein–protein associations. The color of the ring indicates expression level (red, upregulation; green, downregulation). [file BTM2-8-e10346-s001.docx]

**Supplementary Materials**

**Supplementary Methods**

***Transmission electron microscopy of cerium oxide nanoparticles (CNPs)***

The grain sizes of the CNP and interplanar spacing were analyzed using transmission electron microscopy (TEM). CNP was ultrasonically dispersed in 95% ethanol for 10 min. Subsequently, 2–10 μL of the solution was added to a copper grid and air-dried. The grain size distribution of the nanoparticles was studied using a transmission electron microscope (JEM 2010F Microscope, JEOL Ltd., Japan). The interplanar spacing, grain size (n = 10), and selected area electron diffraction patterns of the crystals were measured and analyzed using DigitalMicrograph® version 3 software (Gatan Inc., Pleasanton, CA, USA).

***Hydrodynamic size of CNPs***

The hydrodynamic size of the CNPs was characterized using a Zetasizer NanoZS analyzer (Malvern Instruments, Worcestershire, UK) with dynamic light scattering (DLS). The intensity of the scattered light was detected at 90°, relative to the incident beam. To confirm the dispersion of the synthesized CNPs in different solvents, CNPs were dispersed in double-distilled water, ultrasonically dispersed for 10 min, and measured at 25 °C (n = 3). The obtained data were analyzed using free software (Zetasizer software; Malvern Instruments) provided by the manufacturer.

***X-ray diffraction of CNPs***

An X-ray diffractometer was used to analyze the lattice structures of the synthesized CNPs. The mixture was centrifuged at 9,000 rpm for 30 min, and the supernatant was discarded to obtain the CNP precipitate. The precipitate was placed in an oven at 60 °C for more than 8 h, and the CNP was ground into a powder for X-ray diffraction (XRD) analysis. The phase of the CNP was characterized using TTRAX III (Rigaku, Danvers, MA, USA) equipped with a rotation anode with Cu Kα radiation (λ = 1.542 Å) at 40 kV and 20 mA, scan rate of 0.025°/step at 5 s/step and 2 theta range of 10° to 80°. The obtained data were analyzed using MDI JADE version 6.5 software (Materials Data Inc., Liverpool, CA, USA).

***Specific surface area of CNPs***

The specific surface area was calculated using a particular area of surface analyzer (ASAP2020; Micromeritics, Norcross, GA, USA) by the gas adsorption method. The CNP samples were prepared as XRD CNP samples. The dried CNP powder was degassed at room temperature. The experimental environment was maintained at a temperature of 25 °C and 55% humidity.

***Surface characterization of CNPs***

The surface characterization of CNP was performed using X-ray photoelectron spectroscopy (XPS) (Theta Probe; Thermo Fisher Scientific, Waltham, MA, USA) with an Al K-alpha source to analyze the oxidation state of Ce and evaluate the Ce^3+^/Ce^4+^ ratio. The obtained data were fed into the XPSPEAK41 software^1^ to process peak fitting.

***WST-1 assay***

The WST-1 assay (MK400; Takara, U.S.A), performed according to the manufacturer’s protocol, was used to establish the viability and proliferation of T24 cells upon treatment with 4-HC and CNP. In brief, T24 cells were seeded in 96-well tissue culture plates at a density of 1×10^4^ cells/well and incubated for 24 h for full cell adhesion. To determine the effective concentration of 4-HC, final concentrations of 0, 12.5, 25, 37.5, 50, 75, and 100 μM 4-HC were added to the culture medium for 4 h after full cell adhesion. The cells were washed with PBS and the culture medium was added. Then, 10 μL of PerMix WST-1 solution was added to cells already cultured at 100 μL/well (1:10 final dilution) and light-prohibit incubated for 2 hours at 37 °C in a 5% CO_2_ incubator. The optical density (OD) value at 450 nm was recorded using an ELISA reader (Sunrise Plate Reader; Tecan, Männedorf, Switzerland). The absorbance of the formazan product was measured at 450 nm and detected using an ELISA reader via a colorimetric detection method (n = 6). In this study, the cell viability percentage was calculated using the following formula:

Cell viability (%) = [(OD_sample_−OD_blank_) / (OD_control_−OD_blank_)]×100%.

To determine the effective concentration of CNP, CNP at concentrations of 5, 10, and 25 μg/mL were treated with T24 cells at full adhesion for 24 h. The IC_50_ of 4-HC was then added to the culture medium for 4 h to induce cell damage. The cells were washed with phosphate-buffered saline (PBS) and the culture medium with WST-1 regent was added for another 2 h. The OD value was recorded using an ELISA reader.

***DCFDA cellular reactive oxygen species (ROS) detection assay***

The DCFDA Cellular ROS Detection Assay (ab113851; Abcam, USA) was used to detect intracellular ROS. In brief, T24 cells were seeded in 96-well tissue culture plates at a density of 1×10^4^ cells/well and incubated for 24 h to achieve full cell adhesion.

To determine the effective intracellular ROS concentration in 4-HC, the 4-HC solution was added to the culture medium at final concentrations of 37.5 μM for 4 h. The cells were then washed with PBS, and the culture medium was added to 25 μM DCFDA reagent for another 45 min at 37 °C in a 5% CO_2_ incubator. Finally, the fluorescence intensity was recorded on a Multi-Mode Microplate Reader (SpectraMax i3x; Molecular Devices, USA), and observed and photographed under a fluorescence microscope (IX51; Olympus, Japan)^2^.

To verify the ability of CNP to relieve 4-HC-induced intracellular ROS production, 5 μg/mL of CNP was added to the medium and cultured for 24 h before adding the selected concentration of 4-HC to the cell culture medium for 4 h. The cells were then washed with PBS, and 25 μM DCFDA reagent was added to the culture medium for another 45 min and subjected to incubation. The fluorescence intensity of CNP cells at each concentration was measured in the same manner as described above.

***Rearing activity***

A camera (Sony AS100V Action Cam^3^) was used to note the video of locomotor activity. The highest image capture rate of the camera was 240 frames per second and each captured image had a resolution of 1280×720 pixels. Thus, the camera satisfied the requirement for our study to observe the rearing activity of the mice. The rearing activity was measured for 20 min^4^.

***Voiding spot assay***

The mice were housed in individualized wire mesh-bottomed cages above filter paper (Whatman No.1, AW1001-00917; Sigma-Aldrich, USA) for 3 h^5^, water and food restricted, in a noiseless room on the day of sacrifice (day 14). The filter paper was collected, dried, and imaged using a FluorChem digital imaging system (Alpha Innotech Corporation, San Leandro, CA, USA) under ultraviolet light at 365 nm. The volume was quantified from the area of the spots, in contrast to a calibration curve. The total size area and number of void spots were quantified by inspecting the images using ImageJ with Void Whizzard^6^.

***RNA sequencing***

A total of nine samples derived from nine ICR mouse bladders exposed to various treatment conditions were snap-frozen on carbon dioxide (n = 3) and total RNA was extracted from bladders and cells using TRIzol reagent (Invitrogen, Carlsbad, CA, USA). A total of 1 μg RNA per sample was used as input material for the RNA sample preparations. Sequencing libraries were created using the TruSeq stranded mRNA library prep kit (cat# RS-122-2101; Illumina, San Diego, CA, USA) following the manufacturer’s instructions, and index codes were added to allocate sequences to each sample. Briefly, mRNA was purified from total RNA using poly T oligo-attached magnetic beads and fragmented by heating. First-strand cDNA was synthesized using SuperScript II reverse transcriptase. PCR amplification was performed using 2X PCR Master Mix. After adenylation of the 3′ ends of DNA fragments, adaptors were ligated, and the library fragments were purified with the AMPure XP system (Beckman Coulter, Beverly, USA). The final library quality was evaluated on an Agilent Bioanalyzer 2100 system using DNA High Sensitivity Chips. The libraries were sequenced on an Illumina NovaSeq 6000 platform and 150 bp paired-end reads were generated.

Bases with low quality and sequences from adapters in raw data were eliminated using Trimmomatic (version 0.39)^7^. The filtered reads were aligned to the reference genome using Bowtie2 (version 2.3.4.1)^8^. The user-friendly software RSEM (version 1.2.28) was used to quantify transcript abundance^9^. Differentially expressed genes (DEGs) were identified using EBSeq (version 1.16.0)^10^. Differential gene expression analysis results from the RSEM of individual samples were outlined as fragments per kilobase of transcript per million mapped reads (FPKM), which reports the normalized expression values for a specified gene and clarifies multiple reads for an individual fragment. Transcripts up-and down-regulated relative to controls were recognized as significantly changed only when the false discovery rate (FDR) adjusted *P* value was < 0.05. Relative differences in gene expression were evaluated by comparing the log_2_-fold change (log_2_FC) values between the bladder FPKMs for CYP/control, CNP pre-treatment/CYP, and CNP post-treatment/CYP.

***GO term enrichment analysis using DAVID***

Gene Ontology (GO) term enrichment analysis was performed using Database for Annotation, Visualization and Integrated Discovery (DAVID) v6.7 software package (http://david.abcc.ncifcrf.gov/)^11^. Our specific focus was on GO terms of response to cytokines in the category of biological process (BP) to see if these terms were significantly enriched. GO enrichment analyses of these DEGs were performed using ’clusterProfiler’ package in R using the following parameters: Adjusted *P*-values and *q*-values, both <0.05, and the minimum number of genes enriched for each signal pathway was ≥5^12^.

***Quantibody® array profiling***

Blood was collected from nine ICR mouse bladders exposed to various treatment conditions. Plasma samples were collected from the blood samples extracted by cardiac puncture. The mice were euthanized with CO_2_, and blood was extracted and transferred to EDTA-coated mini vacutainer tubes (BD Biosciences, USA). Blood samples were centrifuged at 3,000 rpm for 10 min at 4 °C within 30 min of extraction, and the plasma was collected, immediately frozen in dry ice, and stored at -80 °C. Nine mouse serum samples (n = 3 in each group) collected in each group were applied to the mouse protein profile array reagent kit (cat. no. QAM-CAA-4000; RayBiotech Life, Norcross, GA, USA) to analyze the levels of multiple protein profiles according to the manufacturer’s recommendations. Briefly, the Quantibody® array, a multiplexed sandwich ELISA-based quantitative array platform, and slide-based antibody array are processed as a sandwich-based immunoassay and for screening and quantitative analysis of protein expression concentration. It utilizes a pair of antigen-specific antibodies to capture the protein of interest on the glass surface. Biotinylated antibodies were used to detect the specific proteins using a laser scanner, InnoScan 710-G (Innopsys; Carbonne, France). Then, the Mapix software (version 8.2.2; Innopsys) was used to analyze the fluorescent signal intensities for individual antigen-specific antibody spots between and among array images and could be utilized to quantitatively analyze the protein expression concentration of each sample. The data were analyzed using the GSM-CAA-4000 data analysis software (RayBiotech Life, Norcross, GA, USA). The levels of differential pro-inflammatory cytokines were measured and compared among the control, CYP, and CNP pre-treatment groups (n = 3 in each group).

***Bioinformatics analysis in patients with UCPPS***

We downloaded the raw microarray gene expression data from the Gene Expression Omnibus website (GEO, www.ncbi.nlm.nih.gov/geo/).^13^ We located two datasets, GSE11783^14^ and GSE28242^15^ for human bladder tissue and urine of non-Hunner type autoimmune cystitis in the NCBI GEO database. The GSE11783 dataset is based on GPL570 (HG-U133_Plus_2 Affymetrix Human Genome U133 Plus 2.0 Array) and includes 10 UCPPS bladder samples (five cases in non-Hunner type lesions) and six normal standard control samples. The GSE28242 dataset is based on the GPL6244 platform (HuGene-1_0-st Affymetrix Human Gene 1.0 ST Array transcript gene version) and includes eight urine samples of UCPPS patients (five cases in non-Hunner type lesions) and five normal control samples.

Subsequently, the raw data were preprocessed with Affymetrix hugene10 annotation data^16^ and Affymetrix HG-U133_Plus_2 array annotation data^17^. Data normalization was achieved based on the robust multiarray average method, including data background adjustment, quantile normalization, summarization, and log base 2 scale transformation^18,19^. Gene expression levels were evaluated using the *Bioconductor* package under an R environment to put out the DEGs via a classical *t*-test. Statistically significant differences in expression levels were determined as a two-tailed *P* value < 0.05, while a significant fold change in genes was defined as logFC > 1. Subsequently, we combined these data to specify the common DEGs in both GSE11783 and GSE28242. The Search Tool for Retrieval of Interacting Genes/Proteins (STRING) database is a free web-based resource and can predict protein interactions^20^. The protein interactions for DEGs were retrieved from the STRING database with protein-protein interaction (PPI) score of > 0.15.

We performed GO term enrichment analysis as mentioned above. Our specific focus was on GO terms of upregulated BP to see if these terms were significantly enriched. Adjusted *P*-values and *q*-values, both <0.05, and the minimum number of genes enriched for each signal pathway was ≥5^12^.

**Supplementary Table S1**. List of primers for qPCR, SerpinB2 siRNA, and negative controls

|  | Sequence (5′-3′) |
| --- | --- |
| Mouse primers |  |
| *α Tubulin* forward | ACACCTTCTTCAGTGAGACAGG |
| *α Tubulin* reverse | CTCATTGTCTACCATGAAGGCAC |
| *Irf7* forward | GGGTGTGTCCCCAGGATCATT |
| *Irf7* reverse | GGGTTCCTCGTAAACACGGTC |
| *Irf9* forward | CCTGCCCATTTCTTCAGCTCC |
| *Ifr9* reverse | TGGTCTTGGCTGCATCGTCC |
| *Il6* forward | ACCCCAATTTCCAATGCTCTCC |
| *Il6* reverse | AACGCACTAGGTTTGCCGAG |
| *Tnfα* forward | GGTGCCTATGTCTCAGCCTCTT |
| *Tnfα* reverse | GCCATAGAACTGATGAGAGGGAG |
| *Hmox1/HO-1* forward | GCCGAGAATGCTGAGTTCATG |
| *Hmox1/HO-1* reverse | TGGTACAAGGAAGCCATCACC |
| *SerpinB2* forward | TTTACAGGCACAAGCAGGAGAT |
| *SerpinB2* reverse | AATCCCCCTGTGGTGTGTTG |
| *Cxcl10* forward | TTCTGCCTCATCCTGCTGGG |
| *Cxcl10* reverse | TTCTCACTGGCCCGTCATCG |
| Human primers |  |
| *GAPDH* forward | AAGGTGAAGGTCGGAGTCAAC |
| *GAPDH* reverse | GGGGTCATTGATGGCAACAATA |
| *Il6* forward | AGTGAGGAACAAGCCAGAGC |
| *Il6* reverse | CAGGGGTGGTTATTGCATCT |
| *Tnfα* forward | ATGGAGACAGATGTGGGGTGTG |
| *Tnfα* reverse | TCCCTGAGTGTCTTCTGTGTGC |
| SerpinB2 siRNA |  |
| Sense | CCAAGGUGCUUCAGUUUAATT |
| Antisense | UUAAACUGAAGCACCUUGGTT |
| Negative control |  |
| Sense | UUCUCCGAACGUGUCACGUTT |
| Antisense | ACGUGACACGUUCGGAGAATT |


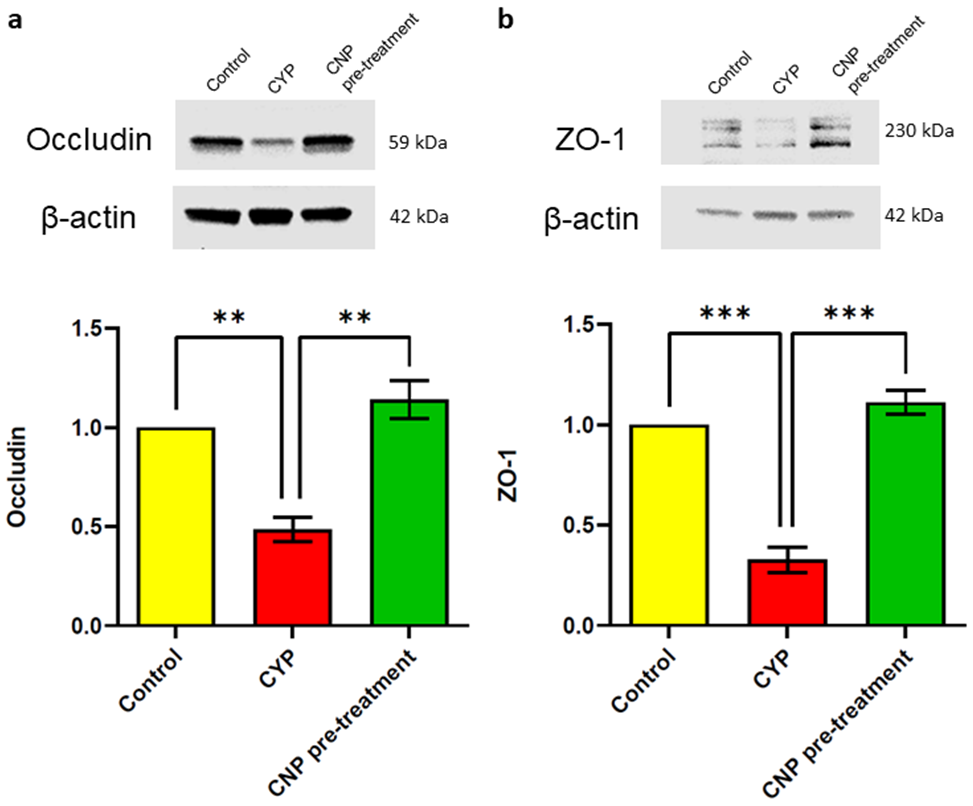


**Supplementary Figure S1**. Expression of tight junction proteins in the bladder. (a) Relative amounts of occludin in the bladders of mice in the control, CYP, and CNP pre-treatment groups. (b) Relative amounts of zonula occludens-1 (ZO-1) in the bladders of mice in the control, CYP, and CNP pre-treatment groups. The values are presented as mean ± standard error. Data were compared using one-way analysis of variance with Tukey’s post-hoc tests. **P* < 0.05, ***P* < 0.01, ****P* < 0.001, n = 3.


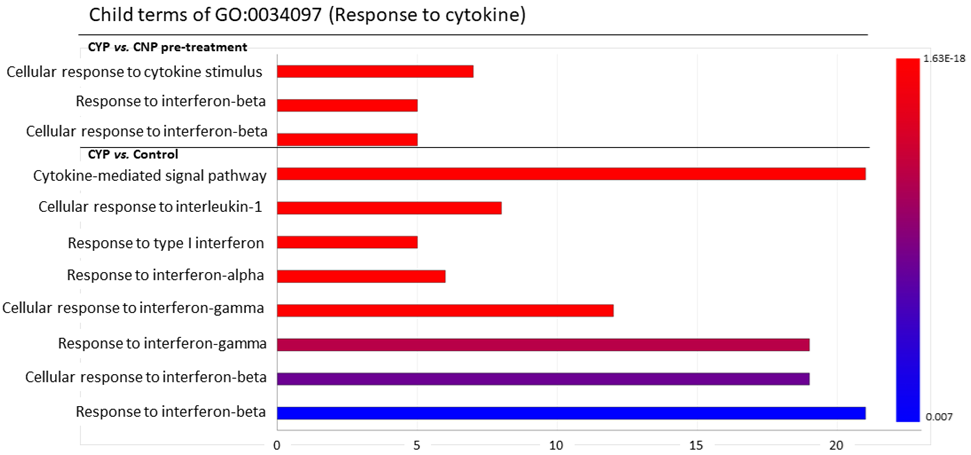


**Supplementary Figure S2**. Enrichment analysis of Gene Ontology (GO) terms of response to cytokines in the biological process (BP) category in the CYP vs. control groups and the CYP vs. CNP pre-treatment groups in the urinary bladders of animals with CYP-induced cystitis. Adjusted *P*-value: Red < purple < blue.


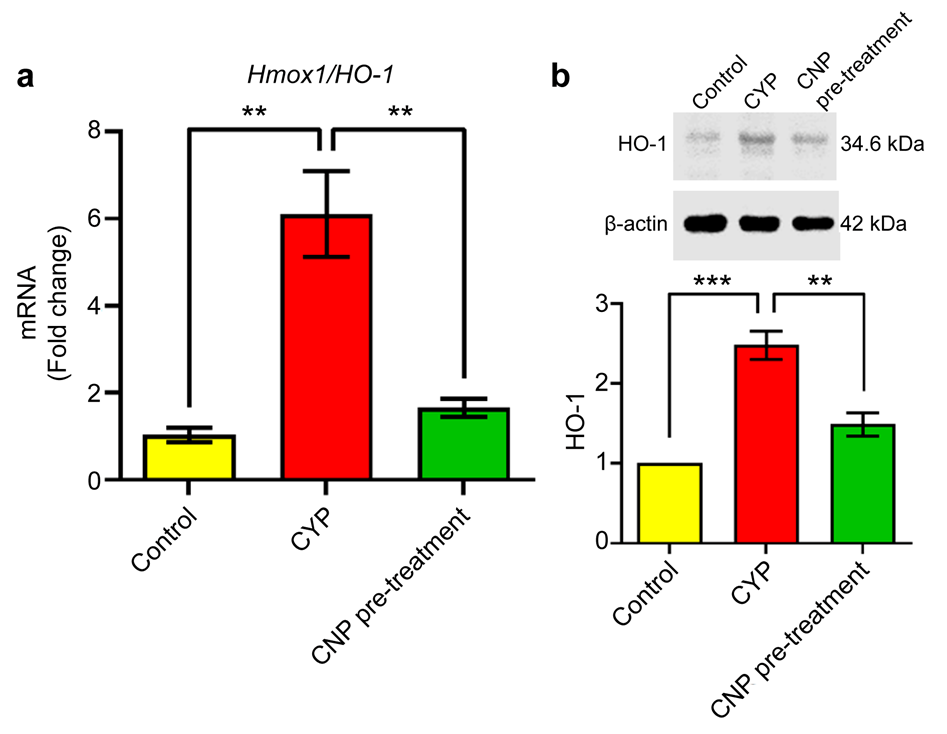


**Supplementary Figure S3**. Expression of heme oxygenase 1 (HO-1) in control, cyclophosphamide (CYP), and cerium oxide nanoparticles (CNP) pre-treatment groups utilizing quantitative real-time polymerase chain reaction (qPCR) assays (a) and Western blotting (b).


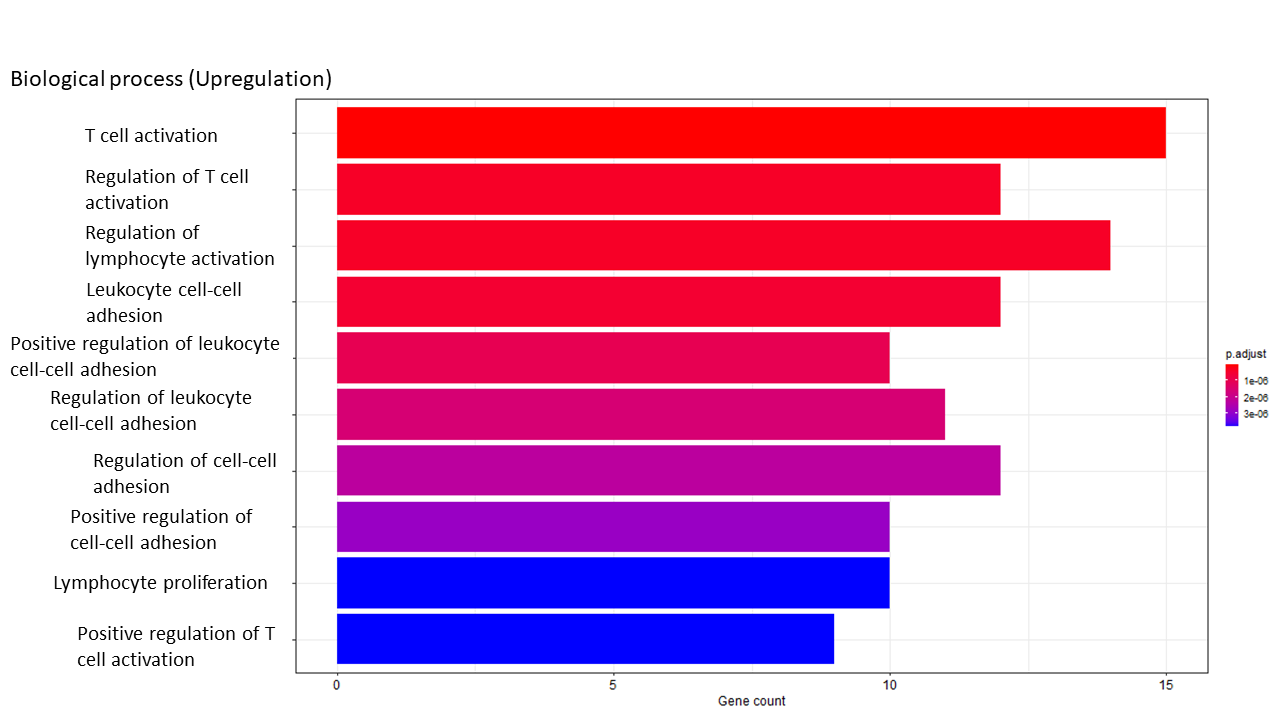


**Supplementary Figure S4**. Enrichment analysis of Gene Ontology (GO) terms of upregulated biological process (BP) in the non-Hunner type cystitis group in comparison with the normal standard control group that collaborated between the human datasets of GSE11783^14^ and GSE28242.^15^ Adjusted *P*-value: Red < purple < blue.


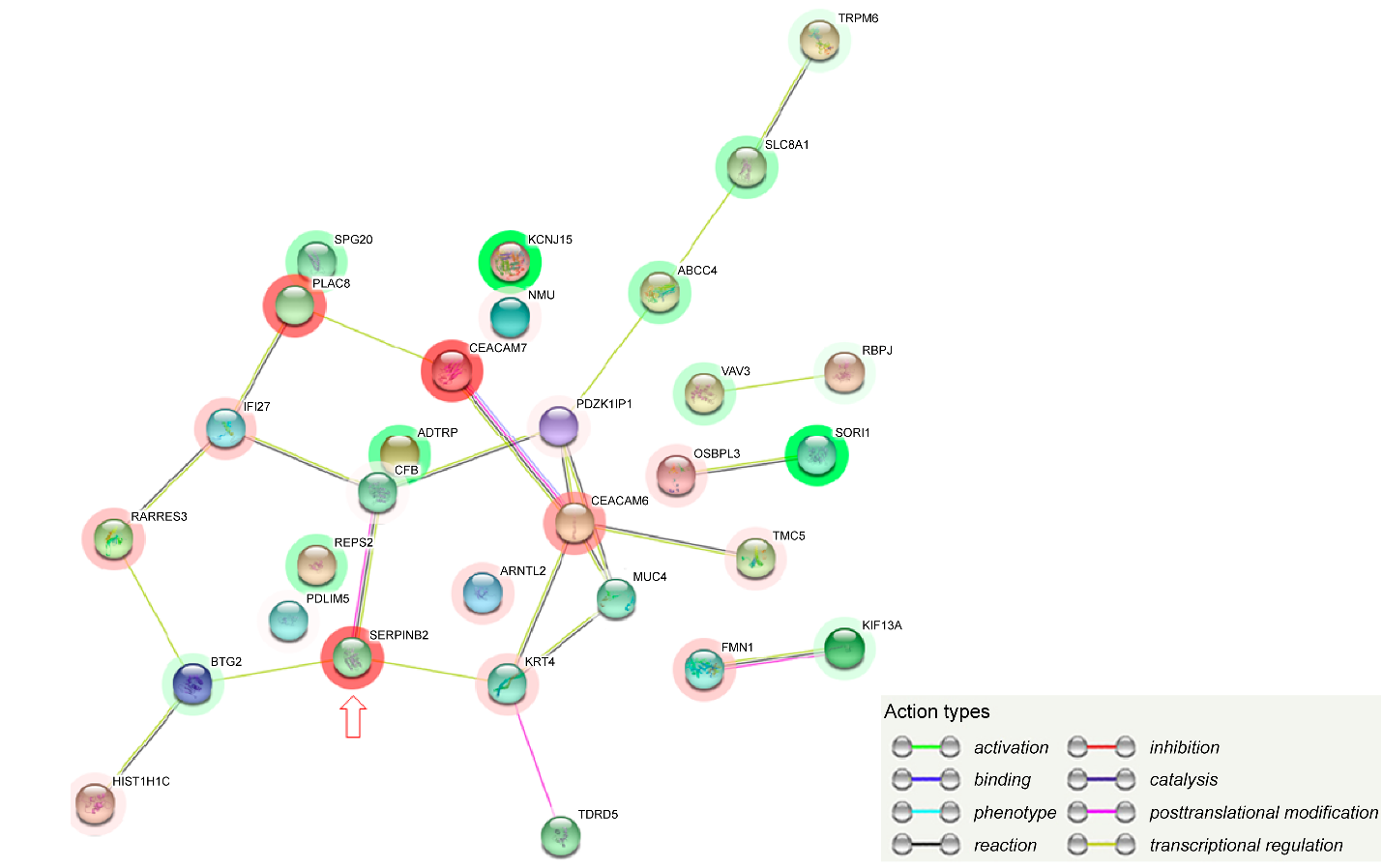


**Supplementary Figure S5**. Protein-protein interaction networks of the 30 differentially expressed genes in the non-Hunner-type cystitis group that collaborated between the datasets of GSE11783^14^ and GSE28242.^15^ Red arrows indicate the location of SerpinB2. Network nodes represent proteins. Edges indicate protein-protein associations. The color of the ring indicates expression level (red, upregulation; green, downregulation).

**References**

1. Walczak, M. S. et al*.* Determining the Chemical Composition of Corrosion Inhibitor/Metal Interfaces with XPS: Minimizing Post Immersion Oxidation. *J. Vis. Exp.* **121**, 55163 (2017).
2. Chou, H. Y. et al. Bifunctional mechanisms of autophagy and apoptosis regulations in melanoma from Bacillus subtilis natto fermentation extract. *Food Chem. Toxicol*. **150**, 112020 (2021).
3. Sony. Digital HD Video Camera Recorder Handbook. (2014).
4. Laird, J. M. A., Martinez-Caro, L., Garcia-Nicas, E. & Cervero, F. A new model of visceral pain and referred hyperalgesia in the mouse. *Pain* **92**, 335–342 (2001).
5. de Oliveira, M. G. et al*.* Deletion or pharmacological blockade of TLR4 confers protection against cyclophosphamide-induced mouse cystitis. *Am. J. Physiol. Physiol.* **315**, F460–F468 (2018).
6. Wegner, K. A. et al*.* Void spot assay procedural optimization and software for rapid and objective quantification of rodent voiding function, including overlapping urine spots. *Am. J. Physiol. Renal Physiol.* **315**, F1067–F1080 (2018).
7. Bolger, A. M., Lohse, M. & Usadel, B. Trimmomatic: a flexible trimmer for Illumina sequence data. *Bioinformatics* **30**, 2114–2120 (2014).
8. Langmead, B. & Salzberg, S. L. Fast gapped-read alignment with Bowtie 2. *Nat. Methods* **9**, 357–359 (2012).
9. Li, B. & Dewey, C. N. RSEM: accurate transcript quantification from RNA-Seq data with or without a reference genome. *BMC Bioinformatics* **12**, 323 (2011).
10. Leng, N. et al*.* EBSeq: an empirical Bayes hierarchical model for inference in RNA-seq experiments. *Bioinformatics* **29**, 1035–1043 (2013).
11. Huang, D. W., Sherman, B. T. & Lempicki, R. A. Systematic and integrative analysis of large gene lists using DAVID bioinformatics resources. *Nat. Protoc.* **4**, 44–57 (2009).
12. Yu, G., Wang, L. G., Han, Y. & He, Q. Y. clusterProfiler: An R package for comparing biological themes among gene clusters. *Omics* **16**, 284–287 (2012).
13. Schroder, W. A. et al*.* SerpinB2 inhibits migration and promotes a resolution phase signature in large peritoneal macrophages. *Sci. Rep.* **9**, 12421 (2019).
14. Gamper, M. et al*.* Gene expression profile of bladder tissue of patients with ulcerative interstitial cystitis. *BMC Genomics* **10**, 199 (2009).
15. Blalock, E. M., Korrect, G. S., Stromberg, A. J. & Erickson, D. R. Gene Expression Analysis of Urine Sediment: Evaluation for Potential Noninvasive Markers of Interstitial Cystitis/Bladder Pain Syndrome. *J. Urol.* **187**, 725–732 (2012).
16. MacDonald, J. hugene10sttranscriptcluster. db: Affymetrix hugene10 annotation data (chip hugene10sttranscriptcluster). R package version 8 (2017).
17. Carlson, M. hgu133plus2.db: Affymetrix Human Genome U133 Plus 2.0 Array annotation data (chip hgu133plus2). R package version 3.2.3. (2016).
18. Bolstad, B. M., Irizarry, R. A., Astrand, M. & Speed, T. P. A comparison of normalization methods for high density oligonucleotide array data based on variance and bias. *Bioinformatics* **19**, 185–193 (2003).
19. Irizarry, R. A. Exploration, normalization, and summaries of high density oligonucleotide array probe level data. *Biostatistics* **4**, 249–264 (2003).
20. Szklarczyk, D. et al*.* STRING v10: protein-protein interaction networks, integrated over the tree of life. *Nucleic Acids Res* **43**, D447-452 (2015).
